# Vicarious ostracism

**DOI:** 10.3389/fnhum.2013.00153

**Published:** 2013-04-23

**Authors:** Eric D. Wesselmann, Kipling D. Williams, Andrew H. Hales

**Affiliations:** ^1^Department of Psychology, Illinois State UniversityNormal, IL, USA; ^2^Department of Psychological Sciences, Purdue UniversityWest Lafayette, IN, USA

Ostracism—being ignored and excluded—causes distress and threatens psychological needs (i.e., belonging, self-esteem, control, and meaningful existence; Williams, 2009). Even subtle behaviors, such as withholding eye contact or staring through someone as if they did not exist, can induce feelings of ostracism (Wirth et al., 2010; Wesselmann et al., 2012). Most individuals experience ostracism at least once in their lives and some experience it daily (Williams, 2009; Nezlek et al., 2012).

Empathy research suggests that individuals vicariously experience others' pain. Most of this research has focused on vicarious physical pain, but might observers also experience vicarious *social* pain (i.e., ostracism)? We will review the emerging research on vicarious ostracism, highlighting the neural correlates of this phenomenon. Finally, we posit future research questions to strengthen the theoretical understanding of vicarious ostracism from social cognitive and evolutionary psychological perspectives.

## Studying vicarious ostracism

We are aware of nine experimental studies demonstrating vicarious ostracism. These studies find that observers recognize an ostracized individual's distress and also feel ostracized themselves (Over and Carpenter, 2009; Wesselmann et al., 2009; Masten et al., 2010, 2011a, 2013a,b; Beeney et al., 2011; Meyer et al., 2012; Will et al., 2013). Vicarious ostracism has been demonstrated in both child/adolescent (Over and Carpenter, 2009; Masten et al., 2010, 2013a,b; Will et al., 2013) and adult samples (Wesselmann et al., 2009; Beeney et al., 2011; Masten et al., 2011a; Meyer et al., 2012; Will et al., 2013). Vicarious ostracism is enhanced when individuals actively perspective-take (Wesselmann et al., 2009), are higher in trait empathy (Masten et al., 2010, 2011a,b, 2013a), or are closely related to the target (Beeney et al., 2011; Meyer et al., 2012).

These studies used two different paradigms. The primary paradigm is Cyberball—a computer-based ball-tossing game in which participants interact with two computer-controlled confederates (Williams, 2009). These confederate players are programmed to either *include* all players equally or to *ostracize* the participant by giving them only two ball tosses at the beginning of the game. Seven studies (Wesselmann et al., 2009; Masten et al., 2010, 2011a,b, 2013a; Beeney et al., 2011; Meyer et al., 2012; Will et al., 2013) adapted this paradigm by programming all of the players' tossing behavior and telling participants they are *observing* a game already in progress. Over and Carpenter (2009) animated two shapes playing ball together. Eventually, another shape approaches the game—this new shape is either similar to the others (ostracism condition) or dissimilar (i.e., a butterfly; control condition). Regardless of condition, the ball-tossing shapes do not toss to the new shape and ultimately avoid the shape.

These studies have impressive diversity in outcome measures. Wesselmann et al. (2009) measured self-reported psychological need threat. Six studies (Masten et al., 2010, 2011a,b, 2013a,b; Beeney et al., 2011; Meyer et al., 2012) used fMRI measures of brain activity in regions associated with experiencing ostracism oneself (Eisenberger and Lieberman, 2004). Four studies using measures of prosocial/affilitative behavior (Over and Carpenter, 2009; Masten et al., 2010, 2011a,b; Will et al., 2013) found that vicarious ostracism increases prosocial/affiliative behavior much like directly experiencing ostracism does (Williams, 2009).

## The neural structure of vicarious ostracism

Observing ostracism increased activity in the dorsal anterior cingulate cortex (dACC) and anterior insula (AI), two brain regions activated by directly experiencing ostracism (Eisenberger and Lieberman, 2004). Observing ostracism also activated the dorsomedial (DMPFC) and medial prefrontal cortexes (MPFC) and precuneus—brain regions associated with *mentalizing* (i.e., thinking about another's mental state; Masten et al., 2011a,b, 2013b). Individual differences in empathy predicted brain activation in both the mentalizing regions (i.e., bilateral DMPFC, MPFC, and temporal parietal junction) and social pain-related regions (i.e., AI and dACC) during vicarious ostracism (Masten et al., 2011a, 2013a). Vicarious ostracism involves different brain regions depending upon the ostracized target; observing a friend's ostracism activated regions associated with direct ostracism experience (i.e., dACC and insula), whereas a stranger's ostracism involved mentalizing-relevant regions (i.e., DMPFC, precuneus, and temporal pole; Meyer et al., 2012). Finally, brain activation in the AI and MPFC—regions associated with trait empathy—correlated with pro-social responses toward the ostracized target (Masten et al., 2010, 2011a).

## Future research questions

### Adaptation?

Researchers have speculated that empathy is an adaptation (Decety and Jackson, 2004). Nairne (2010) argues that an adaptation argument must present evidence that the phenomenon helps organisms survive and reproduce; otherwise the phenomenon could be a byproduct of other psychological adaptations. A compelling case has been made for the survival-relevance of *directly* experiencing social pain (MacDonald and Jensen-Campbell, 2011), but future research should directly test whether vicarious ostracism facilitates differential survival and/or reproduction using evolutionary psychology methods. Otherwise, it is difficult to rule out the possibility that vicarious ostracism only occurs because the neural structures for experiencing both physical and social pain are yoked together (Eisenberger and Lieberman, 2004)?

### Schadenfreude?

In light of the research on vicarious ostracism a paradox emerges: why are rejection/ostracism-based reality television programs (e.g., *Survivor*) popular? When participants are eliminated, they are openly rejected (told they are not wanted; Williams, 2007), and ostracized from the show. Initial rejection undoubtedly hurts, but so does subsequent ostracism; they are no longer on the show, no longer included in activities, no longer talked with or about. These two types of social pain have similar psychological outcomes (c.f., Bernstein and Claypool, 2012). To our knowledge there have been no vicarious *rejection* studies, but based on the extant data on vicarious ostracism and other social pain (e.g., vicarious embarrassment; Krach et al., 2011), it is likely that observing rejection would have similar vicarious effects.

Williams (2009) argues attributions influence interpretations of and reactions to ostracism. Recent neural evidence suggests external attributions for being ostracized (i.e., racism) can help reduce the initial negative effects (Masten et al., 2011b). By extension, an observer's attributions about an ostracized individual may influence vicarious ostracism. Weiner (2006) argues individuals feel satisfaction in others' suffering if perceived as deserved (*Schadenfreude*). Schadenfreude research has found feelings of dislike, anger, or resentment can lead to perceived deservingness and pleasure at another's misfortune, both in self-reports and neurological measures (Weiner, 2006; Feather, 2008; Takahashi et al., 2009). If ostracized/rejected individuals are viewed as deserving their treatment, observers should feel less sympathy for them. Future research should explore if these attributions moderate empathic reactions to ostracism/rejection. Perhaps viewers of rejection-based reality shows lament for individuals unjustly rejected but rejoice when others get what they deserve for behaving anti-socially.
